# Priming With Childhood Constructs Influences Distance Perception

**DOI:** 10.3389/fpsyg.2020.01184

**Published:** 2020-06-30

**Authors:** Hubert Suszek, Mirosław Kofta, Maciej Kopera

**Affiliations:** ^1^Faculty of Psychology, University of Warsaw, Warsaw, Poland; ^2^Department of Psychiatry, Medical University of Warsaw, Warsaw, Poland

**Keywords:** distance perception, priming, childhood memories, regression, self-concept

## Abstract

The aim of our study was to examine whether priming adults with childhood constructs changes distance perception. Two alternative hypotheses could be derived: (1) The fundamental reference approach in visual perception of sizes and distances predicts that priming with childhood constructs should enlarge perceived distance (the world should be larger to a small observer); (2) and, conversely, the action-specific account of perception predicts that priming with childhood constructs should make distances seem shorter (a more physically active child should underestimate distances as more attainable). The results consistently support the second theory. Experiment 1 showed that being either explicitly or implicitly primed with childhood constructs decreased perceptions of distance as compared to that evaluated in the control groups. This effect was noticeable for long distances and only marginally significant for short distances. Also, this effect was not mediated by mood. Experiment 2 replicated the result of explicit priming with an additional control condition (baseline). The effect remained significant after controlling for the participants’ evaluation of their childhood memories, tendency to relive memories from their childhood, having children, having a driver’s license, and the participants’ height.

## Introduction

It is not uncommon for people to report that the objects or spaces they remember from their childhood (such as a room or a school building) look considerably smaller when revisited later in life. This observation has been confirmed in an online study conducted by us recently (see Appendix 1).

These self-reports are in line with the notion that body size serves as a fundamental reference in visual perception of sizes and distances (Warren and Whang, 1987; Bennett, 2011): if body size would indeed play this role, the world should appear larger to a small observer and smaller to a large observer. As a consequence, we might remember objects or spaces from childhood the way they were scaled proportionally to our child body, and now our perception is altered due to the fact that we use the scale of our larger, adult body. Apparently supporting this idea, van der Hoort et al. (2011) found that creating a body illusion altering the participants’ bodies to a tiny size made them perceive objects as larger and farther away. Also, when their bodies were altered to be large, they perceived objects as smaller and nearer when compared with having a normal-sized body. It has also been shown that participants with wide shoulders perceive doorways as narrower when compared to participants with narrow shoulders, and similar differences appear when participants are asked to hold their arms out to their sides (Stefanucci and Geuss, 2009).

Surprisingly, however, results from studies, where distance perceptions in both children and adults were compared directly, contradict the theory discussed so far. Children, particularly those under the age of 10, underestimate distance when compared to adults (Harway, 1963; Da Silva, 1983; Giovannini et al., 2009). Overall, children perceive space as smaller than that perceived by adults. To explain these unexpected findings, we may refer to the action-specific account of perception. According to this, perception is a function of the perceiver’s ability to perform an action, so space is experienced as smaller given that the resources required to cross it are high (Witt, 2011). People dynamically change their perception of the environment depending on their physical resources or the energy costs related to performance needed in a given space: distances were judged to be larger by subjects who were carrying a heavy backpack as compared to those who were not (Proffitt et al., 2003), by older adults when compared to younger counterparts (Sugovic and Witt, 2011), or by participants supposed to throw a heavy ball compared to those who threw a light ball (Proffitt et al., 2003). Also, objects appeared farther away after walking on a treadmill as the effect of an increase in walking effort (Proffitt et al., 2003). Perhaps, then, the fact that children are much more energetic than adults appears to be more important than the fact that they are much smaller in scale. Being full of energy means that your physical resources (relative to energy costs to be incurred) are high; therefore, you should underestimate distances you have to cross.

Vigorous activity seems to be a typical feature of children, and moving seems to be extremely pleasurable to them. Research using accelerometry indicates an almost universal decline in physical activity throughout one’s life span, where childhood (ages 6–11) is the most physically active time in one’s life (Silva et al., 2011; Varma et al., 2017). Thus, the action-specific account of perception implies that it is easier to run for someone who is full of energy and who finds moving pleasurable (a child) than for someone who needs a cup of coffee to be energized and prefers to drive than to walk (an adult). Consequently, a given distance should be shorter to the former person than to the latter.

One may ask whether the action-specific approach to perception may be applied to children. Although there were no studies on children showing directly that action influences distance perception, some findings suggest it indirectly. For instance, Cañal-Bruland and van der Kamp (2009) showed that 5-year-old children who throw balls more successfully to a target perceive the target as bigger. Another study showed that paralyzed children report apparent movement predominantly away from the side of paralysis, which indicates that muscular involvement among children affects perceptual experience (Blane, 1962). Both studies imply that visual perception is intrinsically linked with action intentions, also in children.

The major aim of our study was to examine what would happen with distance perception if one could make adults look at the world from a child’s perspective. Given that we effectively prime a child’s mental perspective in adults, would the distances between them and external objects appear larger or shorter to them?

Previous studies have shown that distance perception could be affected not only by stable age differences but also by transient emotional and categorical primes (Schnall et al., 2005; Stefanucci and Proffitt, 2010). Of particular interest, Chambon (2009) showed that after being primed with an elderly category, young participants estimated distances across a grassy field to be longer than their non-primed counterparts.

Social cognition research has revealed that activation of specific mental constructs can influence particular behavior (Bargh et al., 1996; Smeesters et al., 2010; Weingarten et al., 2016). Constructs associated with the primed representation guide behavior through a direct perception–behavior link (Dijksterhuis and Bargh, 2001). Thus, one can reasonably assume that, just as priming with the elderly category activates the experiential perspective of an old person, priming with the childhood category should activate the experiential perspective of a child, with a perception of space that is specific to this perspective. According to the multiple-self-paradigm (McConnell, 2011), experiences from childhood are organized in the form of distinct idiographic components of the self, like self-schemata (Markus, 1977) or relational selves (Andersen and Chen, 2002). One could therefore expect that activation of the childhood category, saturated with sensual, idiographic material from one’s personal past, could even have stronger psychological effects on distance estimates than activation of the elderly category, since every adult person holds “hot” experiences of him or herself being a child.

So far, the effects of childlike mindset manipulation in adults have only been demonstrated in two studies unrelated to distance perception. It was shown that priming of this kind in adults facilitated creative originality (Zabelina and Robinson, 2010) and promoted prosocial behavior (Gino and Desai, 2012). In our project, we explored how activation of the childhood construct affects distance perception. We anticipated that the notion of childhood could be linked to the bodily experience of being a child. Once adults are primed with their own childhood construct, the associated experiences should be activated through the recollection of their past memories. Consequently, their childlike body characteristics, with accompanying mental state predominant in this life period, should be activated, increasing the likelihood of the occurrence of corresponding behavior.

Previous experiments have demonstrated that people walk more slowly after they have been primed with the elderly category (Bargh et al., 1996; Ku et al., 2010). Supposedly, priming of the childhood category should operate analogously, reinstating body states typical of a child’s experience. As discussed earlier, two alternative hypotheses could be advanced as to how childhood-anchored bodily experiences may affect distance perception: addressing either body size or the energization of behavior (assumedly reducing costs of physical activity). Activating a childlike mental perspective should make distances appear larger according to the first approach and smaller according to the second approach.

## Study 1

In this study, we examined whether priming with childhood constructs would change distance perception. In order to strengthen the validity of the manipulation, two priming procedures were applied: explicit and implicit. In the former, participants were aware of the construct that was being primed; in the latter, they were not. We also controlled for participants’ current mood to check whether mood could mediate the effects. There is a possibility that childhood constructs are associated with a positive affective response which arouses automatically whenever they are activated (see schema-triggered affect: Fiske, 1982). This might be due to the fact that adults rate their childhood as happier (Field, 1981). There is evidence that mood can influence spatial perception. For example, it has been found that observers in a sad mood reported hills to be steeper (Riener et al., 2011).

### Method

#### Participants

Seventy-seven participants (37 females; *M*_*age*_ = 23.51 years, *SD* = 4.29) from the University of Warsaw participated in the study in exchange for small gifts. A sample size was determined using a power analysis using G^∗^Power (Faul et al., 2007) based on the effect size previously obtained by Chambon(2009; Cohen’s *d* = 0.792) who demonstrated that priming with an elderly category altered distance perception. We determined that a sample size of 53 participants would achieve 80% power to detect an effect size of similar magnitude (Cohen’s *f* = 0.396) with an alpha of.05. All participants had normal or corrected-to-normal vision. The study was approved by the Ethics Committee of the Faculty of Psychology at the University of Warsaw, Poland. Informed consent was obtained from all participants.

#### Procedure

The study took place in the garden of the university’s library. The participants were approached by the experimenter and asked to take part in a study on distance perception. The study was introduced as an experiment that consisted of two separate and unrelated parts.

##### Experimental treatment

Participants were assigned at random to four conditions: two experimental groups and two control groups. In the first experimental condition (explicit priming of the childhood category), participants were asked to recall experiences from their childhood and to complete 16 sentences from the perspective of the child they had been at that time. The instructions were as follows: “Please think about the time when you were 7 years old. Please complete the following sentences from the perspective of the child you were at that time.” In the respective control condition, participants were asked to think about their adulthood and to answer analogous questions from their present perspective. All questions are presented in Appendix 2. In the second experimental condition (implicit priming of the childhood category), a scrambled-sentence priming task was used (Srull and Wyer, 1979; Bargh et al., 1996). The task was introduced to the participants as a language processing test, with an apparent aim to investigate how people spontaneously perceive relationships between words and use words in flexible ways. Participants were given 30 sets of five words and were asked to build grammatically correct four-word sentences (e.g., “The sky is blue”). To prime childhood-related concepts, 15 of the sentences contained a childhood-related word (e.g., fun, carelessness, holiday, naive, and crying). In the respective control condition, the sentences contained adulthood-related words (e.g., work, boss, documents, responsible, and cautious). The words were selected based on a previous pilot study.

##### Mood and manipulation checks

After manipulation, participants reported their actual mood on a seven-point one-item scale (1 = *negative*, 7 = *positive*); then, the effectiveness of the manipulation was checked. In the deliberate-manipulation condition, participants answered a two-item manipulation check (“The writing task I completed made me return to my childhood” and “The writing task I completed made me think of what I am like as an adult”) using a 7-point scale (1 = *strongly disagree*, 7 = *strongly agree*). To check if the scrambled-sentence task successfully activated the concept of childhood, in the implicit-manipulation condition, participants were asked to fill in blank letter spaces to make words. Three of the six letter strings could be completed with words related to the concept of childhood (e.g., NA__NY could be completed as NAIWNY or NADANY, which in Polish means either “naive” or “granted”), again selected in a pilot study.

##### Dependent measures

Subsequently, all participants were asked to take part in a distance estimation task, which was the same as that used by Proffitt et al. (2003). Participants judged distances in an outdoor, grassy field. Distances were marked in the field with golf tees, which were not visible to the participants. Participants stayed in the same location throughout the experiment, at the convergence point of the six radii. They had to verbally estimate the distance from themselves to a small orange cone that was placed on a different radius each time. On each trial, participants faced away from the field while the cone was being placed. They then turned around and reported the distance from themselves to the cone. Each participant made 24 distance estimates from 1 to 17 m across six radii (12 practice trials and 12 test trials) presented in a semi-randomized order. The practice trials were intended to encourage participants to adopt a consistent response strategy prior to the test trials. Viewing duration was not limited.

Once finished, the participants answered demographic questions about their age and gender. Finally, the participants completed a funneled debriefing questionnaire (Bargh and Chartrand, 2000) and were fully debriefed.

### Results and Discussion

Two participants were excluded because they expressed their suspicion that both parts of the study were somehow connected.

#### Manipulation Checks

The explicit manipulation was effective. Participants primed with a childhood construct reported that the writing task made them think more about their childhood (*M* = 5.79, SD = 1.03) as compared to the corresponding control condition (*M* = 2.42, SD = 1.68), *t*(36) = 7.46, *p* < 0.001, *d* = 2.42. They also reported that the writing task made them think less about their adulthood (*M* = 2.26, SD = 1.52, *d* = 2.43) as compared to the control condition (*M* = 5.68, SD = 1.29), *t*(36) = −7.47, *p* < 0.001. In the implicit-childhood condition, participants generated more words related to the concept of childhood than those in the corresponding control condition, thus indicating that the task was successful in making the childhood concept more accessible, with the difference being marginally significant: χ(2) = 5.26, *p* = 0.072.

#### Effects of Childhood Prime on Distance Assessments

To examine how priming of a childhood construct affects distance perception, we conducted a 2 × 2 × 2 between-subjects *ANOVA* on distance perception as a dependent measure with priming construct (childhood vs control), type of priming (explicit vs implicit), and gender (female vs male) as factors. The accuracy of perception for each distance was calculated with the following formula: (estimated distance − real distance) / real distance, where negative values represent underestimation and positive values represent overestimation of distance. The general score was calculated by averaging the score from all estimated distances.

There was a significant main effect for the priming construct: *F*(1,67) = 5.03, *p* = 0.028, _*p*_η^2^ = 0.070. Being primed with childhood constructs significantly decreased perceived distances (*M* = −0.116, SD = 0.181 vs *M* = 0.033, SD = 0.369). The main effect of the type of priming was not significant: *F*(1,67) = 1.55, *p* = 0.22, _*p*_η^2^ = 0.023. The effect of gender was not significant either: *F*(1,67) = 2.23, *p* = 0.14, _*p*_η^2^ = 0.032. There were no interaction effects (all *p*s > 0.05). Table 1 summarizes these results.

**TABLE 1:** Analysis of variance and analysis of covariance for distance perception accuracy in Study 1 and Study 2.

Effect	*df*	*F*	*p*	_*p*_η^2^
**Study 1**				
Priming	1	5.03	0.028	0.070
Type of priming	1	1.55	0.22	0.023
Gender	1	2.23	0.14	0.032
Priming × Type of Priming	1	0.028	0.87	0.001
Priming × Gender	1	0.76	0.39	0.011
Type of Priming × Gender	1	0.077	0.78	0.001
Priming × Type of Priming × Gender	1	0.90	0.34	0.013
**Study 2**				
Priming	2	4.45	0.016	0.14
Priming (ANCOVA)	2	4.49	0.016	0.155
Gender	1	0.13	0.72	0.002
Gender (ANCOVA)	1	0.09	0.77	0.002
Priming × Gender	2	1.12	0.33	0.040
Priming × Gender (ANCOVA)	2	0.39	0.68	0.016

*ANCOVA was performed, controlling for an evaluation of childhood memories, the tendency to relive one’s memories from childhood, having children, having driving experience, and the participants’ height.*

It is a well-established finding in the social cognition literature that schemata affect perceptions and interpretations more strongly when target information is uncertain or ambiguous (e.g., Riggs and Cantor, 1984; Carpenter, 1988; Lambert and Wedell, 1991; Sedikides and Skowronski, 1993; Green and Sedikides, 2001; Tuckey and Brewer, 2003). For this reason, in the case of shorter distances (which are easy to assess), the effects of priming with childhood schemata might be relatively weak. However, in the case of longer distances (which are more difficult to assess, judgmental uncertainty is higher), one should expect stronger effects of such priming. To verify this expectation, a three-way repeated-measures *ANOVA* was conducted on distance estimates as a dependent variable with priming construct (childhood vs. control) and type of priming (explicit vs implicit) as between-subjects factors and size of distance (short vs long) as a within-subject factor. The six distances assessed in test trials were divided into three shorter (4, 6, and 8 m) and three longer distances (10, 12, and 14 m). The scores for both categories were calculated with the same formula as for all distances.

**FIGURE 1:**
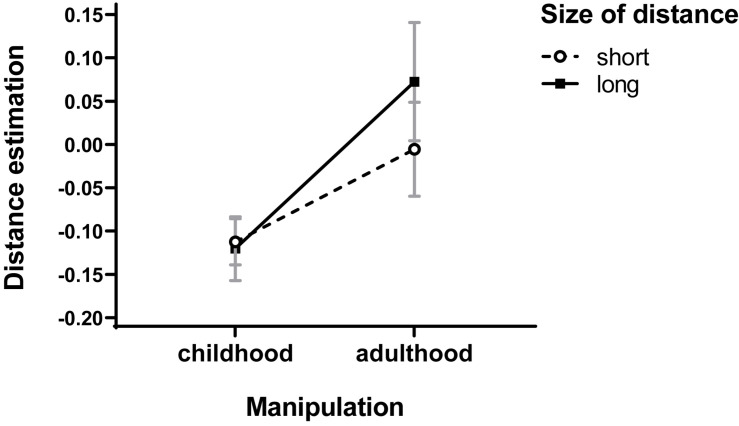
Effects of childhood priming on distance assessment. Error bars represent standard errors of the means.

In addition to the overall main effect of childhood prime on distance perception (the same as the former), this analysis yielded significant interaction of childhood prime × distance size, *F*(1,71) = 4.23, *p* = 0.043, _*p*_η^2^ = 0.056, due to the fact that the effects appeared to be stronger for long than short distances (see Figure 1).

We explored this interaction further. For short distances, a two-way childhood prime × manipulation type *ANOVA* revealed only the main effect of childhood prime at the level of tendency, *F*(1,71) = 3.19, *p* = 0.078, _*p*_η^2^ = 0.043 (*M* = −0.112, SD = 0.159, vs *M* = −0.005, SD = 0.338). For long distances, however, the same effect appeared significant, *F*(1,71) = 6.07, *p* = 0.016, _*p*_η^2^ = 0.079 (*M* = −0.12, SD = 0.221 vs *M* = 0.072, *SD* = 0.426). Thus, results confirm our hypothesis: effects of childhood prime appeared twice as strong for long than short distances.

#### Additional Analyses

To check whether mood could mediate the obtained effect, we first conducted a 2 × 2 × 2 *ANOVA* on self-reported mood with priming construct (childhood vs control), type of priming (explicit vs implicit), and gender (female vs male) as factors. There were no significant main effects for the priming construct, *F*(1,67) = 0.57; *p* = 0.41, _*p*_η^2^ = 0.010; the type of priming, *F*(1,67) = 1, *p* = 0.32, _*p*_η^2^ = 0.015; or gender, *F*(1,67) = 1.48; *p* = 0.23, _*p*_η^2^ = 0.022. Also, no interaction effects were found (*p*s > 0.05). The lack of effects of childhood primes on mood precluded further usage of mediation analysis.

The results showed that priming (explicitly or implicitly) with childhood constructs changed the participants’ perception of distances; that is, the distances were perceived as shorter than in the control groups. These effects were not mediated by changes in mood; perhaps they might have been due to the activation of other features of the childhood frame, for instance, the experience of increased readiness to move in space, thus making objects more “attainable.” Regardless of the explanation given, the effect found in our study contradicts the first prediction (small-body-as-an-anchor theory) and supports the second prediction (the action-specific account of perception theory). Presumably, energy abilities triggered by childhood schemata led the participants to perceive distances as shorter.

## Study 2

Study 1 showed that making participants think about their childhood either deliberately or implicitly led them to perceive distances as shorter than in the control groups. In Study 2, we intended to replicate the findings of Study 1 with a few significant modifications.

As both types of priming were effective, we focused only on explicit manipulation. First, we modified the control group in order to increase the validity of this manipulation. In Study 1, in the control condition of explicit priming, participants were asked to focus on recalling adult experiences from their present. This solution could be criticized on the grounds that the compared conditions differed not only in the content (child experience vs adult experience) but also in the kind of mental set as well (to recall the past vs a present experience). Therefore, in the first control condition of Study 2, participants were also requested to focus on a past that belonged to their adult experience. Also, to make sure that changes in distance perception did indeed occur because of childhood priming (making distances smaller), not because of adulthood priming (making distances larger), we added a second control condition with no manipulation at all (baseline).

Additionally, other variables related to childhood memories were controlled for. Perhaps having “good” or “bad” childhood memories could modify the effects of priming childhood selves on spatial perspective (it is plausible that adverse childhood memories could interfere with the process of activating childhood constructs due to increased risk of reliving the “bad” experiences). Much in the same vein, we also controlled for the potential effects of dispositional aversion or repression of childhood memories on spatial judgments. It has been shown that the inability to remember events from childhood is related to traumatic stress in childhood (Brown et al., 2007). For this purpose, we asked the participants to rate their tendency to relive memories from their childhood. Another potential variable of interest was whether the participants had children, as having children may allow adults to take the childhood perspective more often, so it may become habitual/easier to access.

Also, we took into account two variables that could affect accuracy of spatial judgments. The first was the participant’s height, as it has been shown that taller observers judge distances more accurately than shorter observers (Zhou et al., 2016). Also, we asked whether the participants had a driver’s license and, if yes, for how long (it has been shown that driving experience is related to accuracy in distance perception relative to speed, see Cavallo and Laurent, 1988).

### Method

#### Participants

Sixty participants (30 females; *M*_*age*_ = 25.68 years, SD = 4.57) from the Warsaw University of Life Sciences participated in the study in exchange for small gifts. The power analysis was similar to that of Study 1, suggesting 65 participants. However, due to limits of our subject pool, we only managed to recruit 60 students and thus have an estimated power of 76%. All participants reported normal or corrected-to-normal vision. Five participants (8.3%) declared that they had children, and 37 (67.1%) declared that they had a driver’s license (driving experience: *M* = 4.65 years, SD = 5.39). The study was approved by the Ethics Committee of the Faculty of Psychology at the University of Warsaw. Informed consent was obtained from all participants.

#### Procedure

The study took place in an open space at the university’s park. Participants were asked to participate in a study on memory and distance perception. As was done formerly, the study was described as consisting of two unrelated parts, except for the control group. The childhood manipulation was the same as in the explicit priming condition in Study 1.

The adult control manipulation was changed. Participants were asked to recall experiences from their adult past (to think most of all about the time 1 year ago) and to answer analogous questions from the perspective of the person they were at that time. We added a second neutral control condition without manipulation (baseline).

After treatment, the participants answered the same manipulation check questions as in Study 1 (except for the neutral control condition). Then they had to report on their actual mood (1 = *negative*, 7 = *positive*).

**FIGURE 2:**
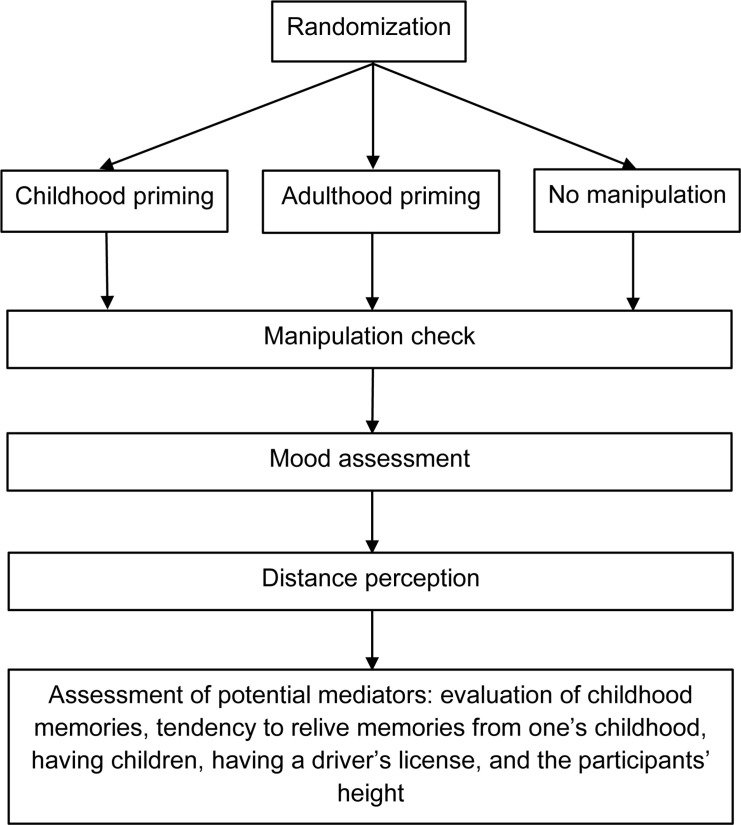
Flowchart for Study 2.

Furthermore, participants answered two questions about their own childhood: (1) “Please estimate to what extent your childhood was happy” (1 = *very unhappy*, 7 = *very happy*) and (2) “Try to estimate how often you think about your childhood” (1 = *almost never*, 7 = *very often*). Also, they answered questions regarding their age, gender, height, whether they had children, whether they had a driver’s license, and, if so, for how many years. Finally, the experimenter conducted a funnel debriefing (Bargh and Chartrand, 2000). Figure 2 shows the flowchart for Study 2.

### Results and Discussion

#### Manipulation Checks

The manipulation check was effective. The participants in the childhood condition reported that the sentence completion task made them think more about their childhood (*M* = 4.65, SD = 2.01) as compared to the adult control condition (*M* = 1.65, SD = 0.74), *t*(38) = 6.27, *p* < 0.001, *d* = 1.98. They also tended to think less about their adulthood (*M* = 4.05, SD = 1.36) as compared to the adult control condition (*M* = 4.95, SD = 1.7), *t*(38) = 1.85, *p* = 0.072, *d* = 0.58, with the difference being marginally significant.

#### Effects of Childhood Prime on Distance Assessments

To examine our main prediction, we conducted a 3 × 2 *ANOVA* with the factors of priming the construct (childhood vs adult control vs baseline) and gender (female vs male) on distance perception, measured in the same manner as in Study 1. This analysis revealed the main effect of priming, *F*(2,54) = 4.45, *p* = 0.016, _*p*_η^2^ = 0.14, due to the fact that participants in the childhood priming condition evaluated distances as being shorter (*M* = −0.201, SD = 0.112) than participants in the adult control condition (*M* = 0.052, SD = 0.415), *t*(57) = 2.879, *p* = 0.006, *d* = 0.83, and shorter than participants in the neutral control condition (*M* = −0.011, SD = 0.217), *t*(57) = 2.166, *p* = 0.035, *d* = 0.58. The effect of gender was not significant: *F*(1,54) = 0.13, *p* = 0.72, _*p*_η^2^ = 0.002. There was no interaction effect: *F*(2,54) = 1.12, *p* = 0.33, _*p*_η^2^ = 0.040. An *ANCOVA* revealed that the effect of priming on distance perception held when controlling for an evaluation of childhood memories, the tendency to relive one’s memories from childhood, having children, having driving experience, and the participants’ height: *F*(2,49) = 4.49, *p* = 0.016, _*p*_η^2^ = 0.155. Again, there was neither significant gender effect, *F*(1,49) = 0.09, *p* = 0.77, _*p*_η^2^ = 0.002, nor childhood prime × gender effect, *F*(2,49) = 0.39, *p* = 0.68, _*p*_η^2^ = 0.016. Table 1 summarizes these results. All of the covariates were insignificant, except for driving experience: *F*(1,49) = 6.24, *p* = 0.016, _*p*_η^2^ = 0.113, which improved accuracy in distance perception.

**FIGURE 3:**
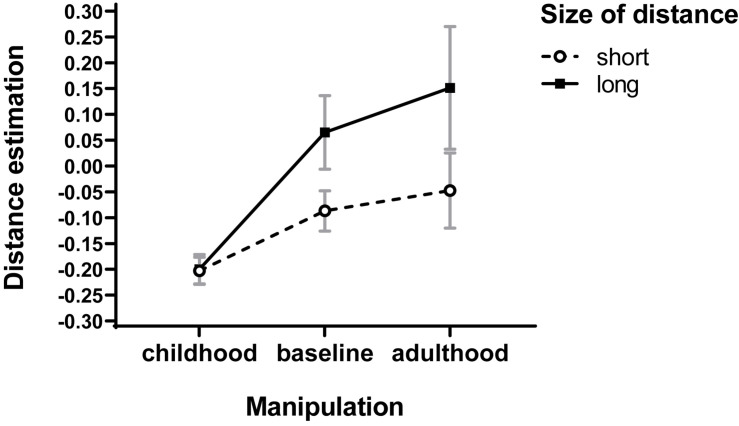
Effects of childhood priming on distance assessment with baseline condition. Error bars represent standard errors of the means.

To examine whether the effects of childhood prime on space perception were modified by short versus long distance, a two-way *ANOVA* was conducted on distance estimates with priming construct (childhood prime vs. adulthood prime vs control) as a between-subjects factor and size of distance (short vs. long) as a within-subject factor. In addition to the overall main effect of childhood prime on distance estimates (the same as the former), this analysis yielded the main effect of distant size, *F*(1,57) = 14.479, *p* < 0.001, _*p*_η^2^ = 0.202 (for short distances, *M* = −0.112, SD = 0.23; for long distances, *M* = 0.005, SD = 0.389), and, as in Experiment 1, a significant interaction of childhood prime × distance size, *F*(2,57) = 3.63, *p* = 0.033, _*p*_η^2^ = 0.113 (see Figure 3), of the same shape as previously found (a stronger impact of childhood prime on long than on short distances).

To explore this interaction further, separate analyses were conducted for short and long distances. For short distances, a one-way *ANOVA* with childhood prime as a factor again revealed the effect of childhood prime at the level of tendency, *F*(1,57) = 2.60, *p* = 0.083, _*p*_η^2^ = 0.084 (*M* = −0.203, SD = 0.118 vs *M* = −0.047, SD = 0.326 vs *M* = −0.087, *SD* = 0.173). For long distances, however, the effect of the same factor appeared significant: *F*(1,57) = 5.02, *p* = 0.009, _*p*_η^2^ = 0.15. *Post hoc* comparisons with a Duncan test revealed that mean distance estimates for the childhood condition (*M* = −0.2, SD = 0.128) were significantly lower than the respective means for the adulthood condition (*M* = 0.151, SD = 0.531) at *p* = 0.005 or baseline condition (*M* = 0.065, SD = 0.318) at *p* = 0.026, with the two latter conditions not differing to a significant degree (*p* = 0.460). Again, the effects of childhood prime (decreased estimates of physical distance) appeared twice as strong for long than short distances. These results are in line with our expectations and replicate the pattern obtained in Study 1.

In the next step, we checked for the effect of mood as potential mediator. We found no difference in mood between the childhood condition (*M* = 5.05, SD = 1.14), the adult control condition (*M* = 5.4, SD = 0.75), and the neutral control condition (*M* = 5.1, SD = 1.52), *F*(2,57) = 0.62, *p* = 0.54, _*p*_η^2^ = 0.021. The lack of effects of the childhood prime precluded the mediating role of mood in the effects of a childhood prime on distance assessment.

To conclude, Study 2 successfully replicated the results from Study 1, showing that after being primed with childhood constructs, participants perceived distances as shorter than in the respective control group. The effect was replicated with an improved control condition (as in the experimental group, participants in this condition were asked to focus on past experience). Second, Study 1 left it unclear whether the effect was indeed due to decreased distances after the childhood prime or, partly at least, to increased distances after the adulthood prime. Therefore, an additional second control condition (baseline, without priming at all) was included in the experimental design. It appeared that it was the childhood prime (not the adulthood prime) that accounted for the effect. Third, the relationship remained significant after the other confounding factors (evaluation of childhood memories, tendency to relive memories from one’s childhood, having children, having a driver’s license, and the participants’ height) were controlled for.

Our basic finding that distances were perceived as shorter in the childhood priming condition than in the control conditions supports the explanation that perception of distance changed as a result of diminished effort activated with the childhood construct.

## General Discussion

In this research, we aimed to explore whether priming with childhood constructs would change distance perception. We assumed that priming with these constructs would activate bodily states characteristic of children. However, the current literature suggests that at least two alternative explanatory frameworks could be advanced, leading to opposing predictions—the first addresses body size and the second the effort needed to move.

The first position assumes that differences in size between the child and adult bodies are critical because body size serves as a fundamental reference in the visual perception of sizes and distances (Warren and Whang, 1987; Bennett, 2011). Therefore, priming a childlike way of experiencing the world should make distances seem larger. This idea is supported by the results of recent studies conducted by van der Hoort et al. (2011), who found that creating the body illusion of being tiny made participants perceive objects as larger and farther away, whereas creating the body illusion of being large made participants perceive objects as smaller and nearer.

The second position, that is, the action-specific account of perception, assumes that space is experienced as smaller given that our resources to cross it are high (Witt, 2011). Undoubtedly, children have more energy resources than adults and are likely to engage in vigorous, pleasurable physical activity. Research utilizing accelerometry supports this common observation—there is a universal decline in physical activity throughout one’s life span, with childhood (ages 6–11) being the most physically active time in one’s life (Silva et al., 2011; Varma et al., 2017). Thus, for a child who is full of energy and who finds moving pleasurable, distances to be crossed should be felt as shorter than for an adult person, who is less energetic and more “lazy.”

Both studies consistently showed that after being primed with childhood constructs, participants perceived distances as shorter than participants in the control groups. In Study 1, we captured this effect by using both explicit and implicit priming; in Study 2, the effect was replicated with explicit priming and more sophisticated control conditions. Moreover, in the latter study, the effect remained significant while controlling for several potentially confounding variables, for example, evaluation of childhood memories, the tendency to relive memories from one’s childhood, having children, having a driver’s license, and the participant’s height. Thus, one may conclude that, indeed, priming the child’s psychological perspective makes perceived distances smaller.

This phenomenon could be better explained by the action-specific account of perception than by the fundamental reference approach. Whereas the latter predicts that priming with childhood constructs should enlarge perceived distance (the world should be larger to a small observer), the former predicts the opposite: priming with childhood constructs should make perception of distances shorter (because the child—being more physically active—tends to see distant objects as more “attainable,” easier to get to). Of course, this hypothesis needs to be examined in future research, in which we measure directly the assumed changes in accelerometer activity after childhood primes and analyze their mediating role in the discussed effect (shortened distance after such prime).

Additional analyses revealed that this effect is noticeable for long distances and only marginally significant for short distances. The finding is in line with what we know about the conditions of the effective of schema priming (assimilation of data to activated schema takes place to the extent that the data are uncertain or ambiguous; see, e.g., Riggs and Cantor, 1984; Carpenter, 1988; Lambert and Wedell, 1991; Sedikides and Skowronski, 1993; Green and Sedikides, 2001; Tuckey and Brewer, 2003). It is more difficult to correctly assess long than short distances just by eye: therefore, activated childhood schema should affect estimates of longer distances more strongly. This finding strengthens interpretation of our results in terms of cognitive schema theory. Also, it makes a methodological contribution: if one would like to test his/her expectations in the domain of physical distance assessments, we would recommend doing separate analyses for short and long distances rather than for aggregated data only.

Overall, the obtained results are consistent with the explanation of distance assessments as proposed by the action-specific account of perception. Assuming this approach, we focus on the fact that children possess high readiness to move, particularly to run. Children see the world as “movers” or “runners”: at high probability, subjective costs of such pleasurable behavior are for them low. Encouraging exploration of the world, their underestimation of distance may be a highly adaptive device that promotes their motor development. Due to the universality of the experience of being a child, these bodily states—encoded in the adult’s memories of childhood—are likely to be reactivated when the child’s mental perspective is primed. As a consequence, such priming alters the ongoing perception of space.

Although the above explanation sounds plausible, it needs corroboration in future studies. As suggested earlier, this could be done by including an objective measure for the energization level. If an action-specific explanation of our findings is valid, then the suppressive effects of the childhood primes on distance perception should be mediated by an increased energization of behavior. Because bodily changes in the “readiness-to-act” state probably occur automatically, that is, without the participant’s awareness, we would suggest that the best way of measuring this mediator would be to use a nonverbal method (e.g., accelerometry). In this context, it seems of utmost interest that our most recent studies (see Suszek, 2019) have indeed showed that priming adults with childhood constructs results in an increased level of physical arousal as indicated by accelerometric performance. Obviously, this finding does not confirm directly the action-specific explanation of the present findings but is in line with the advanced explanation.

In this paper, we assume that priming adults with childhood constructs results in perception of distances as closer due to the fact that it activates the experiential perspective of a child (heightened energization of behavior being its important component). The following question arises: is there any other evidence for this process to occur (in addition to the accelerometric findings just discussed)? One may reasonably assume that “living in the present moment” is a highly characteristic feature of a child’s way of experiencing the world. This kind of time perspective refers to a hedonistic, risk-taking, and pleasure-oriented attitude towards life, with high impulsivity and little concern for the future consequences of one’s actions (Zimbardo and Boyd, 1999). In a series of four experiments (Suszek et al., 2019), we recently found that, indeed, exposure to childhood primes (which were very similar to those used in the present studies) made adult participants more focused on the hedonistic present. This finding provides additional support to an idea that underestimation of distances—resulting after a childhood prime in the present studies—is due to activation of a child’s experiential perspective, with focusing on what is going on here and now, pleasure-seeking, and heightened energization of behavior as its essential features. Obviously, future studies would be necessary to examine whether the effects of childhood primes on space estimates could indeed be attributed to activation of a hitherto-described, children-specific, general experiential perspective.

An argument which has often been raised against the validity of findings supporting the action-specific approach to perception is that such effects could be products of demand characteristics (e.g., Firestone, 2013). However, based on results of our Internet survey about people’s experiences of distances in childhood, which was reported in the introduction, one may rather expect that the demand characteristics would lead to effects opposite to those obtained in the present studies. Also, the results of the funneled debriefing procedure in both experiments speak against explanation in terms of demand characteristics. Participants recognized neither the purpose of the studies nor the link between manipulation and distance estimation.

Despite a consistent pattern of results, our studies have some obvious limitations. First, our participants were young adults. It is difficult to predict whether priming with childhood constructs among older adults would lead to the same effects. Presumably, the older the person is, the less accessible his/her childhood memories are because of the salience of the adult role. On the other hand, however, contrast between the experiential perspective of older adults and children might be greater, resulting in stronger effects of this kind of priming. In future studies, it would therefore be beneficial to select a more age-differentiated group and to select adults with children of various ages. Also, being exposed not only to one’s own children, for example, having a profession connected with children (e.g., that of a teacher), could play a role and should be controlled for.

To conclude, in the present research, we found that activating childhood constructs results in the underestimation of physical distances. This finding is in line with current research on distance perception and is consistent with the action-specific approach to perception, which claims that individuals perceive their environment in terms of their action abilities (Witt, 2011). It also complements the results obtained by Chambon (2009), who demonstrated that priming with an elderly category reduced the physiological potential of young participants and led them to perceive distances as longer. Our experiments demonstrated that priming with a childhood construct had the opposite effect, leading the adult persons to perceive distances as shorter, which suggests an increase in their physiological potential. Thus, while activation of the elderly category in young people may lead to a simulation of bodily states associated with older people, our manipulation may involve the opposite process, that is, of reexperiencing bodily states typical for childhood. Future research seems necessary to examine this explanation of our findings.

The idea of returning to a childlike way of experiencing the world brings to mind the concept of regression as developed by Sigmund Freud and other psychoanalytic theorists (e.g., “regression in the service of the ego;” Kris, 1952). Here, the process is understood as childhood schema activation resulting in reexperiencing mental and bodily states characteristic of previous developmental stages. Our study is one of a few attempts to experimentally invoke the state of regression. One might speculate that, because of the universality of the experience of being a child, childhood schemata should be rather well developed and complex, as there are many characteristic features of the childlike experience and the social role related to childhood. Future studies could further address this and other effects of the activation of childhood constructs.

## Data Availability Statement

The raw data supporting the conclusions of this article will be made available by the authors, without undue reservation.

## Author Contributions

HS conceived, planned, and carried out the experiments. HS and MiK performed the computations. MiK contributed to the interpretation of the results. HS wrote the manuscript with support from MiK. HS, MiK, and MaK discussed the results, commented on the manuscript, and contributed to the final version of the manuscript.

## Conflict of Interest

The authors declare that the research was conducted in the absence of any commercial or financial relationships that could be construed as a potential conflict of interest.

## Appendix 1

In order to explore how common this experience is, we conducted an Internet survey on 247 students from the University of Warsaw (64% female, *M*_*age*_ = 24.27; *SD*_*age*_ = 8.37), asking them two “yes/no” questions: (1) Did you ever experience that a particular distance or space (e.g., a room) that you saw years later as an adult appeared smaller to you than the way you had remembered it as a child? (2) Have you ever experienced that an object you saw years later as an adult appeared smaller to you than the way you had remembered it as a child? We found that 83.4% of respondents answered “yes” to the first question and 68.8% answered “yes” to the second question.

## Appendix 2

Sentence completion task used for explicit priming. The changes for the adult control condition are presented in parentheses.

1. A distinguishing physical characteristic of mine is…

2. I am a child who… (I am a person who…)

3. My usual mood is…

4. Sometimes I behave…

5. My favorite game is… (What I like the most is…)

6. In my room… (In my apartment…)

7. One of my needs is…

8. At school… (At work/At college…)

9. I am interested in…

10. What I dislike the most is…

11. When I get older I would like to… (My ambition is…)

12. My family…

13. My happiest time…

14. My friends…

15. My problem is…

16. I dream about…
